# The validity of Posttraumatic Stress Disorder Checklist for DSM-5 (PCL-5) as screening instrument with Kurdish and Arab displaced populations living in the Kurdistan region of Iraq

**DOI:** 10.1186/s12888-018-1839-z

**Published:** 2018-08-16

**Authors:** Hawkar Ibrahim, Verena Ertl, Claudia Catani, Azad Ali Ismail, Frank Neuner

**Affiliations:** 1 Department of Psychology, Clinical Psychology and Psychotherapy, Bielefeld University, Bielefeld, Germany; 2 vivo international, Konstanz, Germany; 3 Department of Clinical Psychology, Koya University, Koya, Kurdistan Region of Iraq Iraq

**Keywords:** PCL, PTSD, Validation, Arab spring, Displaced people

## Abstract

**Background:**

The Posttraumatic Stress Disorder Checklist (PCL) is a valid and reliable self-report measure for the assessment of Posttraumatic Stress Disorder (PTSD). Recently the PCL was updated according to the DSM-5 criteria for PTSD. So far only a few studies have examined the psychometric properties of the PCL-5, and all of these are restricted to populations living in industrialized countries. The aim of this study was to determine the psychometric properties and diagnostic utility of the PCL-5 as a screening instrument for war-affected displaced Kurdish and Arab populations. The specific goal was to determine a contextually valid cut-off score for a probable diagnosis of PTSD.

**Methods:**

The PCL-5 was translated into Arabic and two Kurdish dialects. Trained interviewers administered these translations as assisted self-reports to 206 adults living in camps for displaced people in Iraq, together with depression and war-exposure instruments. Two weeks later, 98 randomly chosen subjects were reassessed by expert clinical psychologists. In the absence of a gold-standard instrument with proven validity in this context, the expert interviewers applied the PCL-5 items in the form of a clinical interview and used a DSM-5-algorithm to determine a diagnosis of PTSD. Receiver operator characteristics (ROC) were performed to determine a valid cutoff-score.

**Results:**

The internal consistency of the PCL-5 was high (alpha = .85) and the instrument showed an adequate convergent validity. Using the cut-off score of 23, the PCL-5 achieved the optimal balance of sensitivity and specificity (area under the curve = .82, *p* < .001; sensitivity = .82, specificity = .70).

**Conclusions:**

Given that the comparison of the two assessments included both a re-test interval and validation by different interviewers, our results indicate that the PCL-5 can be recommended as an assessment and screening instrument for Kurdish and Arab populations.

## Background

Current humanitarian crises are commonly related to civil wars or large-scale natural disasters. Such catastrophes threaten not only the safety and physical integrity of the affected populations, but also their mental health. A key challenge for an evidence-based response is the identification of cases with psychological distress who need assistance to avoid negative long-term outcomes [1]. One of the global hot spots of insurgencies and war is the Middle East region. Since the Arab Spring, which started as a revolutionary wave of demonstrations in the Arab world in 2010 in Egypt, Libya, Yemen, Bahrain, and finally Syria [2], the region has experienced a period of political instability and civil war. The civil war in Syria and Iraq forced many Syrians and Iraqis to flee, either within their own countries or to Lebanon, Jordan, Turkey, and the Kurdistan Region of Iraq (KRI) as well as to Europe [3, 4]. Although it is very likely that this crisis has had a significant impact on the mental health of the affected populations, studies addressing the effects on mental health arising from this disaster are still scarce. So far, only a few studies have systematically addressed the psychological consequences of the civil war and migration that followed the events of the Arab spring. Consistent with previous studies from other war regions, these investigations documented high rates of posttraumatic stress disorder (PTSD) [5–7]. Studies on mental health and violence in Arab and Kurdish populations have applied translated versions of standardized western instruments for the assessment of violence and trauma related disorders although the cut-off-score of these instruments have not been adjusted in the local context [8]. This procedure is critical, since, due to inevitable differences in subtle semantic nuances, the transference of a cutoff score across contexts and populations even with well-translated instruments may lead to considerable over- or underestimations of prevalence rates [9, 10].

In the past decades a range of assessment and screening tools for trauma and related symptoms, including self-report questionnaires and different types of interview methods have been developed (for reviews see [11, 12]). The PTSD Checklist (PCL) [13] is one of the most widely applied self-report measures for assessing PTSD in clinical and research settings. Recently the PCL was updated according to the new diagnostic criteria for PTSD in the Diagnostic and Statistical Manual of Mental Disorders, Fifth Edition (PTSD Checklist for DSM-5; PCL-5) [14]. The PCL-5 contains twenty items rated on a five-point Likert-type scale, with scores ranging from “Not at all” (0) to “Extremely” (4), resulting in a symptom severity score between 0 and 80. A preliminary version of the PCL-5 suggested a cut-off score of 33 for a diagnosis of PTSD, while validation studies recommended a variety of cutoff scores ranging between 28 and 37 [15–17] or following the DSM-5 diagnostic algorithm for PTSD with items that correspond to the DSM criteria. The findings of validation studies indicate that the optimal cut-off score depends on the context, the population as well as the gold-standard instrument applied in the validation studies.

The aim of this study is was estimate the psychometric properties and diagnostic utility of the PCL-5 as a screening instrument. This study sought to determine an appropriate cut-off score with the optimal balance of sensitivity and specificity for Arab and Kurdish populations affected by the Syrian and Iraqi civil wars. For several reasons, this task is far more challenging than the translation of a psychometric instrument for different European populations, and the established standards for such procedures can’t be transferred to this population. The most obvious complication is that people living in the northern regions Syria and Iraq speak a variety of languages and it is often impossible to determine a main language spoken within this region. Even more, even single subjects living in these regions have difficulties to define their individual main, first or native language, since different languages may be spoken in different contexts. For example, many Kurdish rely on regional dialects for the communication within the families but have been educated in Arabic and refer to this language as soon as they talk to educated professionals or authorities. The individual skills in reading and writing Arab and Kurdish depend on ethnicity but also on the educational level and the specific community of origin. At the same time, different languages have been associated with specific war parties, and some individuals are reluctant to use a certain language as they feel reminded to confrontations with the enemy. Particularly in mixed populations such as in refugee camps and camps for internally displaced people in KRI it is impossible to predict the specific individual language skills and preferences of the single respondents. As a consequence, we refrained from determining a specific language for each population, but let each single respondent chose the individual preferred language for this specific context. Since a substantial proportion of the inhabitants are illiterate or lacks the skills to fill in a questionnaire, we trained local interviewers who were fluent in Arab and Kurdish to administer the Kurdish or Arab translations of the PCL in the form an assisted self-report. In absence of a valid gold-standard instrument available in Arabic or Kurdish, we used clinical interviews carried out by clinically experienced Master’s or PhD-level psychologists from Koya University in KRI, who are fluent in Arabic and Kurdish, as a comparison. We hold that a clinical assessment carried out by experts who are familiar with the local culture and language, and also well educated in international perspectives in trauma research, would present a standard with the highest face-validity in this context. There was no alternative to this solution, since at the time of data acquisition there was no validated clinical interview schedule for the DSM-5 for PTSD in English.

## Method

### Participants

The participants were Iraqi IDP- and Syrian refugee couples who had fled to Arbat Camps in the Sulaymaniyah Governorate in the KRI as a result of the civil war and attacks by rebel armies. Data were collected in two waves and included a full study sample and a validation sub-sample. Participants in the full sample included 108 Iraqi and 98 Syrian displaced persons with diverse religious backgrounds and ethnicities. A random sub-sample consisting of 98 individuals (49 couples) was re-interviewed to determine the reliability and validity of this sub-sample (validation sample). Table 1 summarizes basic demographic characteristics of both samples.

**Table 1:** Sociodemographic information and traumatic experiences

	Full study sample	Validation sample
Interview language N (%)		
Kurdish	148(71.8)	57(58.16)
Arabic	58(28.2)	41(41.84)
Gender, N (%)		
Male	102 (49.5)	49 (50)
Female	104 (50.5)	49 (50)
Age, mean (SD)^a^	32.79 (10.11)	32.85(10.37)
Religion, N (%)		
Muslim – Sunni	154(74.8)	79(80.6)
Muslim – Shia	14(6.8)	3(3.1)
Yazidi	38(18.4)	16(16.3)
Ethnicity, N (%)		
Kurd	143(69.4)	70(71.4)
Arab	54(26.2)	28(28.6)
Other	9(4.4)	0(0)
Nationality, N (%)		
Iraqi	108(52.4)	47(48)
Syrian	98(47.6)	51(52)
Formal education, mean (SD)^a^	6.38(4.35)	6.02 (4.21)
Occupation, N (%)		
Household	117(56.8)	57(58.2)
Full-time work	24(11.6)	9(9.2)
Part-time work	50(24.3)	23(23.5)
Student	7(3.4)	3(3.1)
Unemployed	6(2.9)	5(5.1)
Receiving benefit	2(1.0)	1(1)
Having regular income, N (%)		
No	172(83.5)	82(83.7)
Yes	34(16.5)	16(16.3)
Number of children, mean (SD)	3.88 (2.97)	3.88(2.86)
Length of stay (or time period) in camp as a refugee, mean (SD)^a^	2.63(1.07)	2.68(.93)
Traumatic Experiences		
War-related event types experienced during displacement, mean (SD)^b^	4.28(1.88)	4.62(2.37)
War-related event types experienced life time, mean (SD)^c^	5.26(2.79)	5.87(3.30)
Traumatic event-types experienced, mean (SD)^d^	3.32 (2.67)	2.97(2.47)
Traumatic event-types witnessed, mean (SD)^e^	5.43(3.57)	3.32(2.50)

^a^in year. ^b^score range: 0–11. ^c^score range: 1–17. ^d^score range: 0–15. ^e^score range: 0–17

### Procedure

Local interviewers conducted the screening interviews, and the validation interviews were conducted by expert interviewers. Between December 2016 and January 2017, we recruited six local interviewers (three men and three women). The local interviewers were fluent in Kurdish and Arabic and they had at least a Bachelor’s degree in psychology or social work. Each interviewer attended a one-week intensive theoretical and practical training course on the study instruments. Due to the absence of reliable census data from the refugee camps, we used a pragmatic sampling approach based on a random selection of individuals and households. The camp was sub-divided according to approximately equal household and population size. Local interviewers were assigned to the resulting zones and instructed to randomly select a sampling direction by spinning a pen from the zone center. The first household with one distance to another was selected and from each household, only main householder couples were interviewed.

Our study is part of a much more extensive and cross-national project, which aims to study psychosocial consequences of migration among Iraqi IDPs and Syrian refugees. In the current study, we interviewed displaced Iraqi and Syrian people. We began with a background questionnaire, followed by a war-related events checklist and Life Events Checklist for DSM-5 (LEC-5) [18]. Psychopathology was assessed using the PCL-5 and the depression section of the Hopkins symptom checklist [19]. Participants were fully informed about the procedures of the current study through a standardized informed consent, which included information about aims of our study, confidentiality, potential risks and discomforts, the right to withdraw without prejudice, benefits, and data protection. Verbal informed consent was given, and interviewers documented informed consent for each participant. The interviewers were matched in gender to the interviewees and they were asked about their readiness for re-interview by different interviewers. All participants (except three couples, who had moved to a new location) assented to a further interview. Two weeks later forty-nine couples between 18 and 67 years of age (48% Iraqi and 52% Syrian) were chosen randomly for re-interview by four expert clinical psychologists (two women and two men).

The expert interviewers had at least a Master’s degree in clinical psychology and more than four years clinical experience with highly vulnerable populations including survivors of war, displacement, torture, genocide, and family and gender-based violence. All clinical psychologists were university lecturers at the department of clinical psychology at Koya University in the KRI, and they partially worked as psychotherapists at Koya university’s outpatient clinic. This clinic offers psychological diagnostics as well as counseling and psychotherapy for individuals with different mental health problems in including trauma and PTSD.

About 15 days after the first interview, the expert interviewers conducted validation interviews based on the same instruments. However, the experts were instructed to ask the questions of the PCL in the form of a structured clinical interview. For every single PTSD symptom listed in the PCL5, the clinical experts asked about symptom’s presence and it’s occurrences over the past month. They were instructed to explore as much information as needed about the intensity, relevance, and frequency of each symptom to be able to judge the clinical significance of each symptom. We perceived that this procedure was the best approximation to culturally sensitive structured interviews that have been recognized as a standard gold for diagnosing PTSD.

Clinically significant symptoms were rated at least as “2 = Moderate”. Expert diagnosis of PTSD was then determined using the DSM-5 algorithm, counting all symptoms rated ‘two or more’ as a present. The clinical psychologists were fluent in Kurdish and Arabic languages, and they were blind to the results of the screening interviews. The ethical review committees of Bielefeld University in Germany and Koya University in the KRI approved all study procedures.

### Instruments

#### Sociodemographic information

The first part of the interview comprised a number of questions about basic demographic variables (e.g., gender, age, marital status, income, employment status, individual and household characteristics etc.) and information related to migration.

#### War-related event checklist

On the basis of previous war exposure scales, e.g. the Violence, War and Abduction Exposure Scale [9] as well as focus-group interviews with war-affected refugees and IDPs living in Iraq, we developed the War Exposure Scale (WES), a specific checklist to assess 13 typical war events before, during, and after migration, which reflected the Kurdish and Arab individuals’ traumatic war-related experiences. The respondents were asked if they had ever experienced a specific type of event. The war exposure score was determined as the sum of “Yes” responses to specific event types.

#### Lifetime traumatic events

For assessing potential lifetime traumatic events, the Life Events Checklist for DSM-5 (LEC-5) [18] was used. The LEC-5 is a self-report measure that consists of 16 potential lifetime traumatic events (with one additional item for assessing any other extraordinarily stressful event that not captured in the first 16 items) in four response categories (1) direct experience. (2) witnessing the trauma. (3) learning that a traumatic event has happened to close family member or close friend. (4) experiencing a traumatic event as a part of the daily job or as a first responder (e.g., paramedic, and police). In the present study, only first 16 event items with the first two response categories “direct experienced” and “witnessed” were analyzed. Participants reported the presence of the events during their lifetime with “Yes” and “No” answers.

#### PTSD

The PTSD checklist for the DSM-5 (PCL-5) [14] was used to estimate the severity of PTSD symptoms as well as to establish the presence of a probable diagnosis of PTSD according to the DMS-5. Previous validation studies showed good psychometric properties for evaluating PTSD [15, 17, 20].

#### Depression

Depressive symptoms were recorded on the 15-item depression section (DHSCL) of the Hopkins Symptom Checklist [21]. The DHSCL is one of the most widely used tools for assessing depressive symptoms in cross cultural research [22]. Perceived severity of depressive symptoms in the week preceding the interview was recorded for each symptom on a 4-point Likert-type scale ranging from 1 to 4. The overall mean of the scale reflects depressive symptom level. The internal consistency of DHSCL in the validation sample as well as in full study sample was high (α = .87 and α = .83, respectively).

Instruments were developed and adapted following recommended procedures in transcultural research [23]. Instruments were translated following the guidelines of van Ommeren et al. (1999) [24]. These involve a translation, lexical back translation, blind back translation, as well as focus group discussions with a group of local bilingual experts and a group of study participants, to gain a semantically equivalent consensus translation. Using this procedure, all study instruments were translated into the Kurdish dialects Sorani and Kurmanji as well as into Arabic.

### Data analysis

The statistical package for the social sciences (SPSS) version 24.0 was used for data analysis. Internal consistencies of the PCL-5 total score and its subscale scores were indicated by Cronbach’s alpha. The stability and validity of the PCL-5 total symptoms score, and the subscale scores were calculated using the Pearson correlation coefficient. To determine the correspondence of the local and expert interviews in PTSD diagnosis, Cohen’s kappa was used for different cut-off scores. Sensitivity and specificity coefficients were used to quantify the diagnostic utility of the PCL-5 [25]. Receiver operator characteristics (ROC) were performed to determine the optimum cutoff score by examining patterns of sensitivity and specificity compared to expert ratings. ROC is a graphical plot that visualizes the performance of a binary classifier (sensitivity on the y-axis and 1-specificity on the x-axis) and the area under the ROC curve quantifies the overall performance of a diagnostic test [26]. Examining the relationship between predictor variables of PTSD and PCL-5 score allowed us to investigate the convergent validity.

## Results

### Internal consistency

Cronbach’s α coefficients calculated for the PCL-5 scores of the full study sample and the validation sample showed high alpha values (α = .85, α = .86 respectively). In the validation sample, Cronbach’s α values for PCL-5 sub-scales (intrusion, avoidance, negative alterations in cognition and mood, and hyperarousal symptom clusters were α = .76, α = .88, α = .74, and α = .71, respectively.

### Cutoff score

**Fig. 1:**
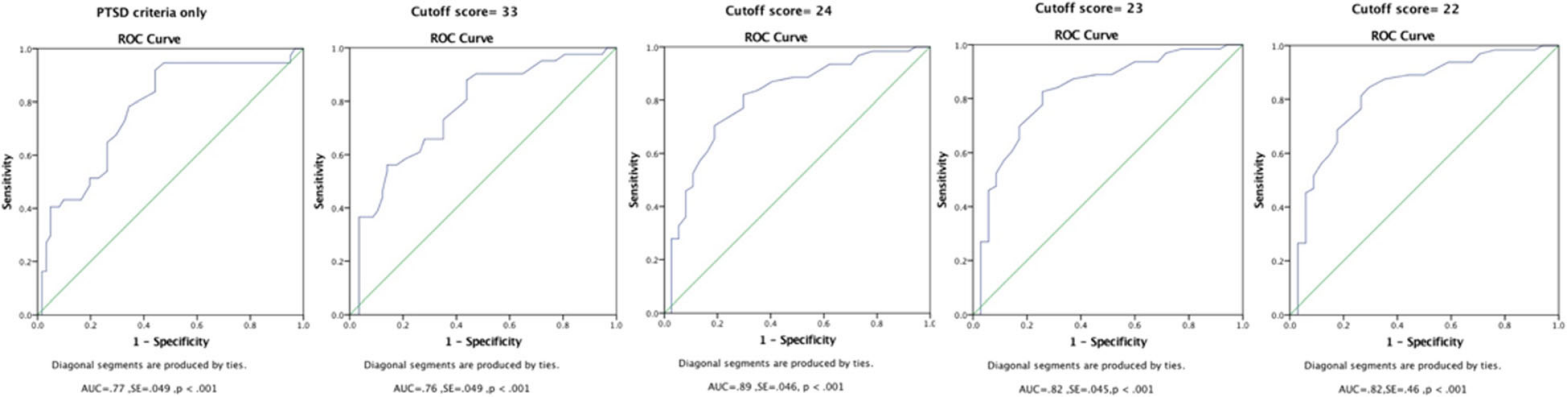
Receiver operating characteristics curves (ROC curve) of the PCL-5 using different cutoff scores. AUCs shows under the curve

Receiver operating curves (ROC) analyses were carried out to determine the optimal cutoff score for the PCL-5 among the Kurdish and Arab populations. ROC is a commonly used method to visualize the sensitivity and specificity of a diagnostic test. ROC curves allow the identification of the best cut-off score for the test by determining the maximum of the area under the curve (AUC; [27]). As presented in Fig. 1 and in Table 2, the PCL-5 reached highest-level balanced sensitivity, specificity and Cohen’s kappa values at cutoff score of 23.

**Table 2:** Performance of the PTSD diagnose by expert interviewers in relative to local interviewers using PCL-5 sum score

Statistic	PTSD criteria only	Cutoff score = 33	Cutoff score = 22	Cutoff score = 23	Cutoff score = 24
Sensitivity	.486	.46	.73	.82	.65
Specificity	.833	.877	.76	.70	.81
ODP	.66	.71	.74	.73	.73
Kappa	.266	.41	.48	.48	.47
*p* value	.007	.000	.000	.000	.000
Prevalence (%)	37.75	41.83	65.30	64.28	62.24

PTSD criteria only = DSM-5 diagnostic algorithm for PTSD. *ODP* overall diagnostic power. Cutoff score of 33 is an initial cutoff score [14]

### Convergent validity

To evaluate the convergent validity of the PCL-5 in the context of this study, we examined the relationship between known predictor variables of PTSD (e.g., war-related and life events) and the PCL-5. Diverse types of traumatic events were significantly positively correlated with the PCL-5 sum score. Moreover, we found a significant positive correlation between symptoms of PTSD (PCL-5 total score) and depression (DHSCL score (Table 3).

**Table 3:** Correlations between the PCL-5 and related constructs (Pearson’s r)

Variables	PCL-5 sum score
War-related event types (during displacement)	.25**
War-related event types (life time)	.23**
Event types experienced	.27**
Event types witnessed	.29**
DHSCL- sum score	.65**

PCL-5 = Posttraumatic Stress Disorder Checklist for DSM-5. *DHSCL* Depression section of Hopkins Symptom Checklist

**. *P* < 0.01, two-tailed

## Discussion

To evaluate the validity and diagnostic accuracy of the PCL-5 in the context of the civil war in Syria and Iraq, we calibrated the PCL-5 cut-off score to the assessment by experienced local clinicians. A ROC curve analyses determined the optimal cut-off score of 23 as marking the optimal balance of sensitivity and specificity. Consistent with previous research [16, 20, 28], we found a high internal consistency of the PCL-5 (alpha = .85). In addition, results showed convincing indications for the convergent validity of the PCL-5. Several types of traumatic events were positively correlated with PTSD symptoms, and this finding is in line with a large number of studies in post-conflict settings [29, 30].

Our findings are only partly consistent with previous validation studies of the PCL-5. The cutoff score in the present study is lower than the initially recommended value and it is lower than the empirically determined cut-off scores from previous validation studies, for instance; using CAPS as a gold standard instrument among military veterans, Bovin et al. (2016) [28] determined an optimal cut-off score of 31. Blevins et al., (2015) [16] evaluated the psychometric properties of the PCL-5 among undergraduate students who identified themselves as having experienced a “very stressful life event” and found that the PCL-5 achieved an appropriate sensitivity, with high specificity and efficiency at a cutoff cut-off score of 37. Similarly, Ashbaugh et al. (2016) [15] studied the psychometric properties of the English and French versions of the PCL-5 among undergraduate students and determined 31 as the optimal score, achieving a sensitivity of.85 and a specificity of.95. However, with the exception of the Bovin et al. (2016) study, these investigations are limited by the fact that other self-report measures were applied as standards.

Our results indicate that the PCL-5 and its contextually validated cut-off score has good psychometric properties as a screening instrument for identifying people with PTSD symptoms among Kurdish and Arab populations. However, it seems that the psychometric values obtained in our evaluation, in particular specificity and sensitivity are slightly lower than those reported in previous validation studies. In contrast to other validation studies we could not rely on a highly structured and reliable gold-standard instrument such as the CAPS, but applied interviews by local experts as standards. It is quite likely that this procedure ultimately reduced the reliability of the standard which, in turn, brought about lower sensitivity and specificity values. In our study, the validation interviews were carried out by experts with a Kurdish and Arabic educational and cultural background, with experience in working in the challenging context of the war region. It is possible that this background has an impact on their evaluation of the thresholds for clinical significance of symptoms of psychological disorders, which may deviate from the standards of a psychiatrist or psychologist from a high-income country. This fact may have contributed to the determination of a lower threshold for clinical significance of a disorder. It is possible that in a challenging and threatening life-context and a hardly functional mental health system lower levels of symptoms lead to dysfunction than in a safe and less threatening environment. In this way, the lower cut-off value of the PSC-5 found in our study might depend on the context of a war-affected community.

The most striking difference is, however, that we determined a lower cut-off value for a potential PTSD diagnosis than in the original study. Several reasons might explain this finding. Possibly, the diagnosis arrived by the local experts in our study was more liberal than in the original validation study, since the decision about the clinical significance of each item did not depend on both, severity and frequency characteristics of each item (as requested in the CAPS that had been used as standard in previous studies) but on the estimation of the clinical importance of each item based on the frequency information as well as the judgement of the expert. Another explanation of this difference might be based on subtle semantic differences in wording that occurred in the translations and tended to result in a meaning that implied a more severe presentation of symptoms in the screening. While, based on the data, we can only speculate about the potential reasons we have to emphasize that, ultimately, any judgment of the clinical significance of a symptom depends on individual clinical expertise as a standard, for example in the validation of the CAPS itself. The involvement of clinical experts with other cultural backgrounds may lead to more discrepant but not necessarily to less valid findings. Future studies using different gold-standard evaluations in different cultures will have to show whether, in general, the recommended cut-off value is too low or if specific linguistic, cultural, or contextual factors contribute to the specific cut-off value in this population.

The results of our study are limited by the absence of a gold standard measure for diagnosing PTSD in Arab and Kurdish populations. While our procedure seems face-valid, it should be complemented by other possibilities, e.g. using standardized translations of established instruments, such as the CAPS-5, once validations of this instrument in English are available. Beyond a clinical interview and predictor variables, future studies should also test if the instrument predicts negative outcomes in mental health and general functioning, as an ultimate test of the performance of a mental health screening instrument. In addition, future studies evaluate should the factorial structure and measurement invariance of PCL-5 in larger samples across different nationalities, ethnic groups, and languages. Another limitation of our study is that we tested three translations of the PCL-5 simultaneously. Although we included almost 100 participants, our sample size was not large enough to allow the comparative evaluation of the single language versions. These limitations are an inevitable compromise reflecting the fact that multiple languages are present in a small region, and that even for single individuals, the preferred language is often not easy to determine. Many respondents spoke one language in their homes but another in the school and obtained different skills and a different vocabulary in the specific languages. Follow-up validation studies should include more distinct populations in other regions to determine the validity in specific populations.

## Conclusions

The current study provided psychometric properties and the diagnostic utility of one of the most widely used screening measure for assessing PTSD as a screening instrument. Our study is the first validation study in the languages of the populations affected by one of the most severe current humanitarian crises. This study provides a potential foundation for further investigations into mental health and trauma in Arab and Kurdish refugee populations as well as a tool for the screening of affected individuals by local health services.

## Abbreviations

DHSCL: Depression section of the Hopkins Symptom Checklist
KRI: Kurdistan Region of Iraq
PCL: Posttraumatic Stress Disorder Checklist
PTSD: Posttraumatic Stress Disorder
